# Optic disc morphology in unilateral branch retinal vein occlusion using spectral domain optical coherence tomography

**DOI:** 10.1186/s12886-015-0165-1

**Published:** 2015-12-12

**Authors:** Andrea Szigeti, Miklós Schneider, Mónika Ecsedy, Zoltán Zs Nagy, Zsuzsanna Récsán

**Affiliations:** Department of Ophthalmology, Semmelweis University, 39 Mária Str., 1085 Budapest, Hungary

**Keywords:** Branch retinal vein occlusion, Optic nerve head, Optic disc size, Cup-disc ratio, Spectral domain optical coherence tomography

## Abstract

**Background:**

The aim of this study was to evaluate the association between optic nerve head (ONH) parameters and branch retinal vein occlusion (BRVO) using spectral domain optical coherence tomography (SD-OCT).

**Methods:**

Both eyes of 40 patients with unilateral BRVO (mean age: 67.4 ± 11.4 years, male: female - 18:22) were enrolled in this study. Control group consisted of randomly selected single healthy eyes of 40 age and gender matched volunteers (mean age: 64.7 ± 15.4 years, male: female - 16:24). ONH parameters (including optic disc area, optic cup area, neuroretinal rim area, cup volume, rim volume, cup-disc area ratio, horizontal and vertical cup-disc ratio, average retinal nerve fiber layer) were measured by SD-OCT. Axial length (AL) of the eyes was measured by non-contact optical low coherence reflectometry. The ONH parameters of eyes with BRVO were compared with those of fellow eyes using mixed model, one-way between-groups analysis of covariance was conducted to compare the ONH parameters of affected and unaffected fellow eyes in BRVO patients with those of the control eyes keeping confounding factors, including AL, age and gender under control in the statistical analysis.

**Results:**

None of the investigated ONH parameters of affected BRVO eyes, unaffected fellow eyes and control eyes were statistically different after controlling for AL, age and gender.

**Conclusion:**

Optic disc morphology might not be a potential anatomical predisposing factor for development of BRVO.

**Electronic supplementary material:**

The online version of this article (doi:10.1186/s12886-015-0165-1) contains supplementary material, which is available to authorized users.

## Background

Branch retinal vein occlusion (BRVO) is the second most frequent retinal vascular disease with a prevalence rate of 0.3–1.1 % [1].

The major risk factors for BRVO include age, hypertension, coexisting cardiovascular diseases and retinal precursor signs (e.g., focal retinal arteriolar narrowing, severe arteriovenous nicking, smaller arterio-venous ratio) [1–3].

Morphological characteristics of the optic nerve head (ONH) are well known features of some ocular diseases. Patients with optic nerve head drusen have smaller optic disc size [4]. Smaller disc area and cupping are predisposing risk factors for the development of nonarteritic anterior ischemic optic neuropathy. [5] The optic disc area is significantly larger in eyes with high myopia compared with those of emmetropic or hyperopic patients [6].

Evidence for direct ONH parameter changes in BRVO eyes is still inconclusive and controversial [7–15]. It has been hypothesized that patients with RVO have smaller discs and narrow scleral canal, which may play a role in increased intraneural tissue pressure, leading to greater susceptibility of vein occlusion [7].

Spectral domain optical coherence tomography (SD-OCT) is a commercially available device with high reproducibility in ONH parameters and RNFL thickness measurement in eyes both of healthy individuals and glaucoma patients [16, 17].

In this study, we aimed to compare quantitative topographic ONH parameters of patients with unilateral BRVO with age-gender matched control subjects using SD-OCT.

## Methods

### Subjects

This prospective controlled study was carried out at the Department of Ophthalmology, Semmelweis University, Budapest, Hungary. All participants were treated in accordance with the tenets of the Declaration of Helsinki. Institutional Review Board approval was obtained for all study protocols (Semmelweis University Regional and Institutional Committee of Sciences and Research Ethics). Written informed consent was obtained from all participants.

Forty patients with unilateral BRVO (mean age: 67.4 ± 11.4 years, range: 40–83 years, male: female - 18:22) were enrolled consecutively as they were referred to the outpatient clinic of the department for examination between January 2013 and January 2015. Mean duration of symptoms was 6.5 months (range: 2 to 12 months). (Additional file 1: Table S1)

The control group consisted of 40 randomly selected healthy eyes of 40 age and gender matched volunteers (mean age: 64.7 ±15.4 years, range: 40–95 years, male: female - 16:24). Right eye was randomly selected in 21 patients (53.0 %) and left eye in 19 patients (47.0 %). (Additional file 2: Table S2)

BRVO was classified according to their anatomical location either as major, when one of the major branch veins draining one of the retinal quadrants was occluded, or as macular, when only one of the smaller venules within the macula was occluded [18]. Twenty-nine patients (72.5 %) had major BRVO and 11 patients (27.5 %) had macular BRVO. Twenty-five patients (62.5 %) had superotemporal BRVO and 15 (37.5 %) had inferotemporal BRVO. Right eye was affected in 19 patients (47.5 %) and left eye in 21 patients (52.5 %).

According to previous studies [19, 20] primary open-angle glaucoma (POAG) is a risk factor for the development of RVO. Glaucomatous optic disc changes may confound the relationship between BRVO and ONH parameters, therefore criteria for inclusion were: intraocular pressure <22 mmHg, no evidence of glaucomatous changes in optic disc or no history of POAG or anti-glaucoma treatment and no history of POAG in first-degree relatives. Patients with a history of previous intraocular surgery, eye trauma, any other retinal or neurological disease (e.g. multiple sclerosis), intraocular inflammation or tumor, or significant ocular media opacities including dense cataract that precluded optical AL measurements were excluded from the study. We also excluded BRVO patients who had optic disc swelling.

Exclusion criteria were the same for control participants with the addition of presence or history of BRVO. All patients and control subjects were Caucasian.

All patients underwent a comprehensive eye examination, including best corrected visual acuity (BCVA, measured with Snellen chart adjusted at 5 m, converted to logMAR values for analysis), subjective manifest spherical equivalent refraction (SER), intraocular pressure measurement with applanation tonometry, slit-lamp biomicroscopy, gonioscopy, and indirect ophthalmoscopy following pupil dilation, and fundus fluorescein angiography in BRVO patients.

SER was defined as the spherical power plus half of the minus cylindrical power (sphere+ ½ cylinder). Axial length (AL) of the eyes was measured by non-contact optical low coherence reflectometry (LenStar LS 900® Haag-Streit AG, Koeniz, Switzerland, software version: V1.3.0). All patients with BRVO underwent systemic examinations, including fasting blood glucose level determination, systemic blood pressure measurement and detailed cardiovascular and hematological examination.

### Spectral domain optical coherence tomography (SD-OCT) measurements

A commercially available RTVue-100 SD-OCT (Optovue Inc., software version 6.9.0.27) was used to measure the ONH parameters and average peripapillary retinal nerve fiber layer (RNFL) thickness. The device uses a laser diode with 840 ± 10 nm wavelength captures 26000 A-scans/second, it has 5 μm axial and 15 μm transverse resolution. For our measurements the ONH scan protocol was used which consists of 12 radial scans of 3.4 mm in length (452 A-scans each) and 6 concentric ring scans ranging from 2.5 to 4.0 mm in diameter (587 to 775 A-scans each), all centered on the optic disc. [17, 21].

The software delineates the optic disc margin by identifying and joining the retinal pigment epithelium / Bruch’s membrane (RPE/BM) tips. Because of improper identification of RPE/BM endpoints may affect the ONH parameters and the retinal nerve fiber layer (RNFL) measurements, the software's automatic detection of the disc margins with 24 RPE/BM endpoints were reviewed and if necessary, corrected manually to ensure proper centering over the disc before analysis [17, 22, 23].

The ONH protocol calculates various parameters that describe the ONH. The optic cup is automatically defined by the RTVue software as the intersection points of the nerve head inner boundary and a parallel line that is 150 μm above the connecting line of the RPE/BM tips [21]. ONH parameters measured by the software included optic disc area, optic cup area, neuroretinal rim area, cup volume (V), rim volume, cup-disc area ratio (CDaR), horizontal cup-disc ratio (hCDR), and vertical cup-disc ratio (vCDR). The ONH protocol also generates a polar RNFL thickness map, measured along a circle 3.45 mm in diameter centered on the optic disc, and gives the average RNFL thickness along the entire measurement circle. Only images with a scan score index (SSI –signal strength index) of more than 35 were included in our study. The SD-OCT operator was masked for clinical findings.

### Statistical analysis

Statistical analysis was performed using SPSS software program (Statistical Package for Social Sciences, SPSS version 22.0; SPSS Inc., Chicago, IL, USA). P value of <0.05 was considered statistically significant.

Differences between demographic data of BRVO patients and control group were assessed by Chi-square test for categorical variables (gender, presence of hypertension and diabetes mellitus) and independent *t*-test for continuous variables (age).

The distributions of SER, AL, ONH parameters (optic disc area, optic cup area, neuroretinal rim area, cup volume, rim volume, CDaR, hCDR, vCDR and average RNFL) were confirmed as normally distributed by Kolmogorov-Smirnov tests and therefore SER, AL, ONH parameters of the eyes with BRVO were compared with those of the unaffected fellow eyes using paired *t*-test. SER, AL, ONH parameters of the affected and unaffected fellow eyes of BRVO patients were compared with those of the control eyes using independent *t*-test.

According to previous study results, ONH parameters correlates with gender (disc and rim area were significantly greater in men), positively associated with AL and age [24–27]. In order to compare ONH data between affected and unaffected fellow eyes of BRVO patients linear mixed model was used, keeping AL, age and gender under control. A one-way between-groups analysis of covariance (ANCOVA) was conducted to compare the ONH parameters of affected and unaffected fellow eyes of BRVO patients with those of the control eyes keeping confounding factors, including AL, age and gender under control in the statistical analysis. Preliminary checks were conducted to ensure that there was no violation of the assumptions of normality, linearity, homogeneity of variances, homogeneity of regression slopes, and reliable measurement of the covariate. Model adjusted R square indicates the strength of the model. Partial eta squared value indicates how much of the variance in the dependent variable is explained by the independent variable. Convert the partial eta squared value to a percentage by multiplying by 100.

## Results

The characteristics of the patients are summarized in Table 1. No significant differences were observed between the groups in terms of age, gender and disease factors, including diabetes mellitus and hypertension (*p*>0.05).

**Table 1:** Patients characteristics

Variables	BRVO	Control	*p* values
Number	40	40	
Gender (male:female)	18:22	16:24	0.615*
Age (mean ± SD, years)	67.4 ± 11.4 (40–83)	64.7±15.4 (40–95)	0.371**
Hypertension (n, %)	27 (67.5 %)	24 (60.0 %)	0.485*
Diabetes mellitus (n, %)	7 (17.5 %)	8 (20.0 %)	0.775*

Chi-squared test for categorical variables (*) and independent *t*-test for continuous variables (**)

In the BRVO group, mean BCVA was +0.42 ± 0.40 logMAR in the affected eyes and +0.04 ± 0.11 logMAR in the unaffected fellow eyes. BCVA of control eyes was +0.01 ± 0.04 logMAR. The mean AL of affected and unaffected fellow eyes in BRVO patients were significantly shorter than those of the control eyes (*p*<0.05) (Table 2.).

**Table 2:** SER, AL, ONH parameters (mean ± standard deviation) of the affected and unaffected fellow eyes in BRVO and control eyes

	Control eyes (*n*=40)	BRVO patients (*n*=40)		*p* value		
		Affected eyes	Unaffected fellow eyes	Affected vs control ^†^	Unaffected fellow vs control ^†^	Affected vs unaffected fellow ^††^
SER (D)	0.70±1.41	0.89±1.51	0.95±1.65	0.606	.520	0.757
AL (mm)	23.48±0.68	22.99±0.56	23.09±0.51	0.001*	0.005*	0.052
Disc area (mm^2^)	1.88 ± 0.22	1.84 ±0.30	1.87 ±0.34	0.491	0.873	0.482
Cup area (mm^2^)	0.54 ± 0.31	0.51±0.39	0.55±0.45	0.692	0.911	0.423
Rim area (mm^2^)	1.31 ± 0.30	1.33±0.38	1.34±0.47	0.727	0.697	0.901
Rim V (mm^3^)	0.16±0.07	0.20±0.18	0.18±0.12	0.166	0.338	0.421
Cup V (mm^3^)	0.07±0.08	0.07±0.09	0.09±0.12	0.880	0.511	0.509
CDaR	0.30±0.18	0.27±0.19	0.29±0.22	0.485	0.796	0.492
hCDR	0.55±0.22	0.51±0.24	0.52±0.27	0.402	0.602	0.582
vCDR	0.47±0.16	0.47±0.23	0.46±0.25	0.978	0.907	0.878
aveRNFL (μm)	100.64±7.38	100.86±11.09	101.13±9.26	0.918	0.796	0.879

*P*-values of independent (^†^) and paired (^††^) *t*-test. Asterisk (*) indicates a difference that is considered statistically significant (*p* < 0.05). (Abbreviations: *SER*= spherical equivalent refraction, *AL*= axial length, *V*=volume, *CDaR*= cup-disc area ratio, *hCDR*=horizontal cup-disc ratio, *vCDR*= vertical cup-disc ratio, *aveRNFL*= average peripapillary retinal nerve fiber layer thickness)

Table 2 shows the ONH parameters: the mean optic disc area, optic cup area, neuroretinal rim area, cup volume, rim volume, CDaR, hCDR, vCDR and average RNFL thickness values. No statistically significant difference was found between affected and unaffected fellow eyes of the BRVO group and the control eyes in terms of these parameters (*p*>0.05).

After controlling for AL, age and gender, there was no significant difference in ONH parameters between these eye groups as well using mixed model and ANCOVA (*p*>0.05, Table 3.)

**Table 3:** Model adjusted R square, partial eta squared and p-values of ANCOVA (^†^) and p values of mixed model (^††^)

	Affected vs control^†^			Unaffected fellow vs control^†^			Affected vs Unaffected fellow^††^
	*P*-value	Partial eta squared	Adjusted R squared	*P*-value	Partial eta squared	Adjusted R squared	*P*-value
Disc area (mm^2^)	0.152	0.027	0.059	0.565	0.004	0.022	0.516
Cup area (mm^2^)	0.352	0.012	0.032	0.702	0.002	0.045	0.592
Rim area (mm^2^)	0.660	0.003	0.041	0.543	0.005	0.080	0.735
Rim V (mm^3^)	0.096	0.036	0.054	0.194	0.022	0.088	0.510
Cup V (mm^3^)	0.598	0.004	0.019	0.687	0.002	0.001	0.519
CDaR	0.217	0.020	0.067	0.415	0.009	0.086	0.672
hCDR	0.168	0.025	0.026	0.341	0.012	0.027	0.759
vCDR	0.637	0.003	0.026	0.493	0.006	0.054	0.573
aveRNFL (μm)	0.432	0.008	0.071	0.822	0.001	0.040	0.760

(Abbreviations: *SER*= spherical equivalent refraction, *AL*= axial length, *V*=volume, *CDaR*= cup-disc area ratio, *hCDR*=horizontal cup-disc ratio, *vCDR*= vertical cup-disc ratio, *aveRNFL*= average peripapillary retinal nerve fiber layer thickness)

## Discussion

BRVO is the second most frequent cause of retinal vascular disorder after diabetic retinopathy with an incidence of 0.5 % to 1.2 % [3]. The recent meta-analysis by Rogers et al. confirmed no gender differences but showed that there are ethnic variations with higher incidences found in Asians and Hispanics [1]. Several risk factors, such as age, hypertension, hyperlipidemia, diabetes mellitus, thrombophilia and hypercoagulation, systemic and inflammatory diseases (e.g. systemic lupus erythematosus, sarcoidosis, syphilis, Behçet disease), medications (e.g. oral contraceptive drugs, anabolic steroid abuse) have been found to be associated with BRVO [1, 3]. Demographic data, proportion of hypertension and diabetes mellitus in our BRVO patients were consistent with previous reports in the literature [28–30].

BRVO is predisposed by various ocular factors. According to previous studies [19, 20] POAG is a risk factor for the development of RVO. Incidence of RVO in the Israeli study over 4 years was 1.85 per 1000 in the general population and 17.3 in glaucoma patients. Patients with glaucoma had equal chance of developing BRVO or central retinal vein occlusion (CRVO) [31].

BRVO occurs at an arteriovenous crossing site, where the artery passes anterior to the vein in 97 %. [32] In 66 % of eyes with BRVO, there is an occlusion of the major branch in the superotemporal quadrant followed by 22–43 % of eyes with occlusion of the major branch in the inferotemporal quadrant, probably because of the increased number of arteriovenous crossings in superotemporal quadrant [33, 34].

Lee et al. found an association between retinal vessel and optic disc diameter using stereoscopic photographs of 3887 right eyes of population of Beaver Dam. Smaller optic discs were associated with more narrow retinal venules and arterioles, smaller central retinal artery and vein diameters [35]. This anatomical relationship may indicate that optic disc size plays a role in the development of RVO.

Citirik hypothesized that smaller disc and small physiologic cupping is the consequence of a relatively small scleral canal and a small opening in Bruch membrane or both may contribute to increased intraneural tissue pressure, and may be associated with distortion of the retinal vessels leading to RVO [7]. Optic disc stereometric parameters may play a role in the development of BRVO. Evidence for the association between ONH parameters and BRVO is still inconclusive and controversial.

Previous studies [10, 11] that examined optic disc size by fundus photograps in RVO patients did not find any correlation between the size of the ONH and BRVO. Gusek et al. [11] measured optic discs of 140 patients with CRVO or BRVO using photographs of the optic disc and correcting the photographic enlargement by Littmann’s method. They found that eyes with RVO had optic discs with a statistically normal size. Mansour studied the horizontal and vertical optic disc diameters in eyes of 67 patients with BRVO and in age-gender-refractive error matched control eyes and found no difference either [10].

Ravalico et al. [13] also found no difference in the distribution of CDRs comparing those with BRVO to control subjects in their study. However, the major disadvantage of their study was that only the CDRs of the fellow eyes of patients with BRVO were compared with those of the control subjects.

The Bejiing Eye Study [9] was a population based prospective cohort study of 4439 Chinese people. Color optic disc slides were used for the measurement of the optic disc size. RVO was detected in 60 eyes of 58 subjects, and RVO was statistically independent from the optic disc size. The authors did not categorize RVO patients according to the occlusion site. Using logistic regression the prevalence of RVOs was significantly associated with age and the presence of glaucomatous optic nerve damage [9].

Hayreh et al. [14] graded the unaffected fellow eyes of 1222 RVO patients (768 CRVO, 183 hemi-CRVO, 271 BRVO) according to CDR into 3 groups: no cup, CDR <0.5, CDR ≥0.5 from stereoscopic photographs of the optic disc. The prevalence of CDR ≥0.5 in all CRVO and hemi-CRVO groups was significantly greater compared with controls. The prevalence in major and macular BRVO with ≥0.5 CDR was not significantly different from the controls. In their study, when there was no cup, BRVO prevalence was higher than CRVO or hemi-CRVO. They evaluated only CDR and no other ONH parameters.

Klein et al. [12] found an association of optic disc cupping and RVO in their prospective epidemiological study (The Beaver Dam Eye Study). They reported that individuals with larger cups and larger vCDRs had significantly greater risk of RVO (about 40 % increase for each 0.1 increment in vCDR). In addition, they found that neither disc diameter nor rim area was associated with incident of RVO. Most of their cases were BRVOs [12].

However, the major limitation of all these previous studies is that all optic disc parameters were obtained from stereoscopic or nonstereoscopic fundus photographs and the authors did not measure and involve AL in the statistical analysis. Some of them evaluated only CDRs, and not the disc or cup area, and in some studies the RVO group was very heterogeneous. The evaluation of fundus photographs is subjective and depends on the experience of the examiner. Compared with optic disc stereo photographs, confocal scanning laser tomography and OCT provides objective and quantitative data [7, 16, 17, 36].

Citirik et al. [7] evaluated topographic parameters of the ONH in 35 eyes of 30 patients with BRVO and compared with fellow eyes and age-matched normal control eyes using confocal scanning laser tomography (Heidelberg retinal tomography, HRT). In contrast to previous study results, they found significantly smaller disc area, cup area, rim area, cup volume, rim volume, and mean cup depth in eyes with BRVO. Mean CDR was smaller in eyes with BRVO than in control eyes, but the statistical difference was not significant. Optic disc parameters were significantly different between affected and unaffected fellow eyes or control eyes but were not different between unaffected fellow eyes and control eyes. They did not involve the AL in their statistical tests.

Actis et al. [15] evaluated ONH parameters in patients with different types of RVO using HRT. In their RVO patients, the most frequent occlusion site in the not-glaucomatous patient group was at the level of an arteriovenous crossing. They found a smaller cup area, smaller CDR and higher rim volume in the arterovenous crossing RVO group. They did not measure AL in their study.

The Singapore Indian Eye Study [8] was a population based study of 3400 Singapore Indians aged over 40 years. Optic disc parameters were quantified using HRT. They found 19 BRVO eyes of 18 participants. Eyes with BRVO had larger disc area than eyes without BRVO, no significant differences in other ONH parameters were observed. The frequency of glaucoma was 4.4 % in non-BRVO eyes, none of the BRVO eyes had glaucoma. In the analysis of eyes without glaucoma, eyes with BRVO had larger disc area, cup area, CDaR, hCDR and vCDR compared to eyes without BRVO. BRVO was associated with larger optic disc and cup area (*p*=0.036, *p*=0.029) and larger CDaR (*p*=0.037) after controlling for age, gender, systemic (hypertension, diabetes, myocardial infarction) and ocular factors (intraocular pressure, glaucoma, central corneal thickness, axial length, previous laser photocoagulation). Eyes with disc area >2.1mm^2^, cup area >0.74mm^2^, and CDaR >0.37 had a 5.07, 5.44 and 5.02 increased odds of association with BRVO. The results were very similar in the analysis of eyes without glacuoma.

According to previous studies [30, 37], HRT and RTVue-100 devices were not interchangeable for ONH analysis. RTVue-100 values were larger for the cup area, CDRs, and HRT values were consistently larger than RTVue-100 values for rim area. Basically, the main difference between the two devices is the position of the reference plane in relation to the optic disc margin. The RTVue-100 automatically determines the edge of the ONH as the end of the the RPE/BM layer. A straight line connects the edges of the RPE/BM, and a parallel line is constructed 150 μm anteriorly. Structures below this line are defined as the disc cup and above this line as the neuroretinal rim. In contrast, the HRT machine automatically calculates a reference plane that is located 50 μm posterior to the retinal surface. Structures underneath the reference plane and within the contour line are defined as the disc cup. Structures above the reference plane and within the contour line are defined as the neuroretinal rim [37]. In the present study, we investigated ONH parameters in patients with unilateral BRVO using SD-OCT for the first time. The optic disc area, optic cup area, neuroretinal rim area, cup volume, rim volume, CDaR, hCDR, vCDR, and average RNFL were not found significantly different in eyes with BRVO, unaffected fellow eyes or healthy control eyes.

Many previous studies [38–42] found that AL in BRVO eyes was significantly shorter than those in control subjects. SER of our patients did not differ significantly, but the mean AL of affected and unaffected eyes in BRVO patients were significantly shorter than those of the control eyes. In most previous studies that evaluated ONH parameters in BRVO, ALs of affected or control eyes were not measured [7, 9–12, 14, 15]. Previous studies reported that there was a correlation between ONH parameters and AL, age, gender or even race in healthy eyes [24–26] and for this reason, the effect of AL, age and gender must be taken to consideration in statistical analyses to obtain reliable results.

Before evaluation of ONH parameters using SD-OCT, it is very important to make sure the RPE tip placements (that were automatically determined by software) are in the correct positions (end of RPE/BM at the disc margin), or adjust if necessary. If the optic disc drawing or the RPE tip placement is not accurate, the measurements can be affected [22, 23].

Immediately after the RVO, RNFL thickness can increase because of edema in the occluded area. Kim et al. analyzed longitudinal changes in RNFL thickness in BRVO, they found initially increased and gradually decreasing RNFL thickness and after 6 months the RNFL of the affected eyes was significantly thinner than those of the unaffected fellow eyes [43]. Previous studies evaluated eyes with acute RVOs [15], where sectorial nerve fiber swelling at the optic disc could have resulted in smaller cup measurements and might have influenced other ONH parameter measurements (e.g. vCDR, CDaR). Therefore we excluded BRVO patients with optic disc swelling from our study. Mean RNFL thickness did not differ in affected and unaffected fellow eyes of our unilateral BRVO patients and in age-gender matched healthy control eyes.

Optic disc swelling can cause difficulties in evaluating ONH parameters. First, the edema makes it difficult to detect the RPE/BM endpoints accurately. Second, the damage to the RNFL caused by retinal edema can influence other ONH parameters (e.g. optic cup and rim parameters, CDRs).

There are several limitations to our study. First, we evaluated only the presence of hypertension and diabetes mellitus in our subjects. Other systemic risk factors such as hyperlipidemia, cardiovascular diseases, blood hyperviscosity and diseases with hypercoagulation were not part of the study. Second, the limitation of optical biometry is its inability to measure through dense cataracts and other media opacities that obscure the macula, therefore patients with significant ocular media opacities were not enrolled. Third, we did not use correction for magnification in OCT optic disc measurements. Leung et al. [44] evaluated the relationships between optic disc measurements and AL. The modified AL method derived from Bennet et al. [45] was used to correct the OCT measurements for ocular magnification. The magnification corrected disc area correlated significantly with the AL, for each millimeter increase in AL, the optic disc area increased by 0.095 mm2. The determination of disc area has significant influence in the evaluation of all the optic disc-related parameters. Correction for magnification in OCT optic disc measurements is therefore very important and should not be overlooked. Fourth, POAG is an important risk factor for the development of RVO, glaucomatous optic disc changes may confound the relationship between BRVO and ONH parameters, therefore patients with POAG were not enrolled in this study, but it would be interesting to evaluate the role of POAG in BRVO patients and ONH parameters with multivariate analysis in a larger number of patients.

## Conclusion

In summary, to the best of our knowledge this is the first study to evaluate optic disc parameters in patients with BRVO using SD-OCT. We did not find statistical difference in optic nerve head morphology and quantitative topographic ONH parameters between BRVO eyes, unaffected fellow eyes and age-gender matched healthy control eyes keeping confounding factors, including AL, age and gender under control in the analysis. Optic disc size might not be a potential anatomical predisposing factor for development of BRVO.

## Additional files

Additional file 1: Table S1. Demographical data (age; gender; hypertension, diabetes mellitus) type of occlusion, affected eye, SER, BCVA, AL, ONH parameters and SSI of affected and unaffected fellow eyes of our BRVO study subjects. (XLS 59 kb) Additional file 2: Table S2. Demographical data (age; gender; hypertension, diabetes mellitus) involved eye, SER, BCVA, AL, ONH parameters and SSI of control patients. (XLS 44 kb)

## Abbreviations

SD-OCT: Spectral domain optical coherence tomography
RVO: Retinal vein occlusion
BRVO: Branch retinal vein occlusion
CRVO: Central retinal vein occlusion
ONH: Optic nerve head
AL: Axial length
POAG: Primary open-angle glaucoma
SER: Spherical equivalent refraction
BCVA: Best corrected visual acuity
RPE: Retinal pigment epithelium
BM: Bruch’s membrane
ANCOVA: One-way between-groups analysis of covariance test
V: Volume
CDaR: Cup-disc area ratio
hCDR: Horizontal cup-disc ratio
vCDR: Vertical cup-disc ratio
aveRNFL: Average peripapillary retinal nerve fiber layer thickness
SSI: Signal strenght index
